# Cell competition: Winning out by losing notch

**DOI:** 10.4161/15384101.2014.988027

**Published:** 2015-01-02

**Authors:** Maria P Alcolea, Philip H Jones

**Affiliations:** 1 MRC Cancer Unit; University of Cambridge; Hutchison/MRC Research Center; Cambridge Biomedical Campus; Cambridge, UK

**Keywords:** carcinogenesis, cancer, esophagus, field change, progenitor, squamous, stem cell, EE, Esophageal Epithelium, Nicd, Notch Cytoplasmic Domain, DN-Maml1, Dominant negative mutant of Mastermind like 1, GFP, Green Fluorescent protein, EYFP, Enhanced Yellow Fluorescent Protein, PP, Cell division producing 2 progenitor cells, DD, Cell division producing 2 differentiated cells, PD, Cell division producing one progenitor and one differentiating cell

## Abstract

Cell competition where ‘loser’ cells are eliminated by neighbors with higher fitness is a widespread phenomenon in development. However, a growing body of evidence argues cells with somatic mutations compete with their wild type counterparts in the earliest stages of cancer development. Recent studies have begun to shed light on the molecular and cellular mechanisms that alter the competitiveness of cells carrying somatic mutations in adult tissues. Cells with a ‘winner’ phenotype create clones which may expand into extensive fields of mutant cells within normal appearing epithelium, favoring the accumulation of further genetic alterations and the evolution of cancer. Here we focus on how mutations which disrupt the Notch signaling pathway confer a ‘super competitor’ status on cells in squamous epithelia and consider the broader implications for cancer evolution.

Cell competition is a phenomenon that occurs between cells in a single tissue compartment as ‘winning’ cells with specific phenotypic advantages out compete their less fit neighbors. It was first described and has been extensively studied in *Drosophila,* but more recently has been observed in mammalian development.^1-4^ However, the cellular mechanisms underlying this important process are far less well defined. Competition was originally described in ‘losers’ carrying a mutation which slows cellular proliferation relative to normal cells. More recently, ‘super competitor’ mutants which win over wild type cells by actively promoting their extrusion or apoptosis have been identified.^5-7^ Genes and pathways reported to function as super competitors in *Drosophila* include *dMy*c and *Wg*, *Hpo* and *Stat* pathway mutants, whose homologues are frequently mutated in human cancer.^6,8-14^

To progress into a tumor, an individual mutant cell must generate a clone which persists for sufficient time to acquire additional genomic changes.^15^ Many genetic alterations reduce fitness relative to wild type cells and are eliminated by cell competition, which provides a defense against cancer development in tissues such as the mouse thymus.^16,17^ In contrast, a cell with a somatic super competitor mutation may drive out wild type cells, colonize a region of tissue and persist long term. This process is well illustrated in squamous epithelia, such as the epidermis, head and neck epithelium and esophagus. In these tissues, carcinogen exposure creates mutant clones within normal appearing tissue.^18,19^ These may expand to take over large areas from which multiple dysplastic lesions and squamous cell carcinomas arise as additional mutations occur.^20,21^ The existence of super competitor mutants may offer an explanation for how such areas of ‘field change’ arise.

Studies on cell competition require the ability to label and visualize mutant cell clones. Until recently, such lineage tracing experiments were well developed only in *Drosophila*.^9^ However, advances in transgenic technology, particularly the availability of inducible alleles of *cre* recombinase and reporter strains that allow the recombined cells to be visualized have allowed clonal lineage tracing into mice.^22^ Moreover, by tracking the size distribution of large samples of clones over time it is possible to quantify the behavior of their constituent cells.^23-26^ This approach has been successfully applied to squamous epithelia in the mouse.^17,19,21-23^ Of these tissues, the uniform architecture of the esophageal epithelium (EE), makes it an ideal model to study epithelial cell competition in a mammalian context.

Murine EE consists of layers of keratinocytes (**Fig. 1A**). It is devoid of appendages such as crypts or glands which form a niche for stem cells in other tissues.^24,26-28^ Proliferation is confined to cells in the basal layer. On commitment to differentiation, basal cells exit the cell cycle and subsequently lose adhesion to the underlying basement membrane allowing them to stratify into the overlying suprabasal cell layer.^24^ They then undergo a series of morphological and biochemical changes until they reach the tissue surface from which they are shed. In normal EE, cell production in the basal layer is precisely matched with shedding at the epithelial surface, so cellular homeostasis is maintained. To achieve this, a 1:1 ratio of proliferating and differentiating cells must be generated across the basal layer.^29^

**Figure 1.**
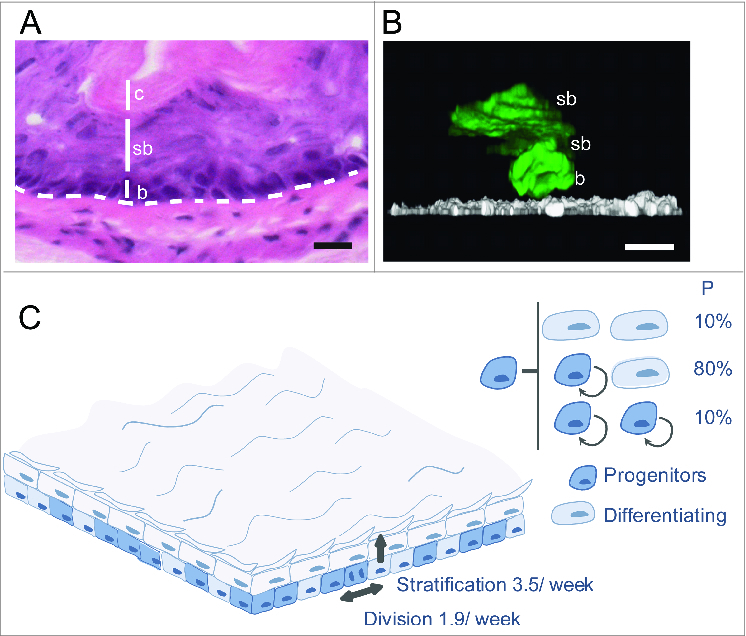
Stochastic cell fate behavior of normal mouse esophageal epithelium. (**A**) Section of mouse esophageal epithelium showing multilayered squamous tissue devoid of appendages. Basal cells (b) overlie a basement membrane (dashed line) above submucosa. Basal cells stratify into suprabasal layers (sb), migrating toward the surface of the epithelium, lined by cornified cells (c), which are continually shed into the esophageal lumen. Scale bar 50 μm. (**B**) Side view of a 3-dimensional reconstruction showing typical EYFP labeled (control) clones 10 d post induction.^24^ EYFP is green and α6 integrin in white, scale bar 10 μm, b indicates a basal cell and sb suprabasal cells. (**C**) Cell fate in normal homeostatic mouse esophageal epithelium.^24^ Progenitor cell division is linked to the exit of a nearby differentiating cell from the basal layer. The average rates of progenitor cell division and differentiated cell stratification are 1.9/week and 3.5 /week respectively. Each division may have one of 3 outcomes: 2 progenitor daughters, 2 differentiating daughters (a terminal division in which neither daughter divides again) or one cell of each type. The outcome of an individual division is unpredictable, but the likelihood of each division outcome, indicated as a percentage, is the same for all progenitors.

Transgenic measurement of cell proliferation in EE reveals all cycling cells divide at the same average rate.^24^ There is no evidence of the slow cycling ‘reserve’ stem cell population described in other tissues.^29,30^ Long-term lineage tracing of a large, representative sample of proliferating cells in homeostatic EE reveals they are a single functionally equivalent population of progenitor cells (**Fig. 1B****,C**).^24^ Stratification of a differentiating cell is linked to division of a nearby progenitor. Progenitor division may have one of 3 outcomes, generating 2 progenitor cells that will go on to divide again, 2 differentiating cells that exit the basal layer and are eventually shed from the tissue, or one cell of each type. The outcome of an individual progenitor division is unpredictable, but the probabilities of each type of division are balanced, with the likelihood of PP and DD divisions being equal (**Fig. 1C**). It follows that across the basal layer, equal numbers of progenitor and differentiating cells are generated, achieving homeostasis.

The single progenitor paradigm differs from models which postulate that a hierarchy of stem and transit amplifying cells maintains homeostasis, proposed on the basis of studies in which basal cells are sorted for cell surface markers and assayed for their colony forming efficiency in 2 or 3 dimensional cell cultures.^31,32^ It is not perhaps surprising that cells differ in their ability to survive isolation by prolonged typsinization and then proliferate in a non-physiological, often growth factor loaded, culture environment.^32^ Marker expression may depend on factors such as whether cells are in a particular phase of the cell cycle or initiating differentiation, that may impact on colony forming efficiency.^33^ Therefore, inferring cell behavior in homeostatic tissue from clonal culture seems unreliable compared with tracking cells in their ‘native habitat’ by lineage tracing.^22^

A consequence of EE progenitor cell behavior is that the progeny of a given cell division have a high likelihood of being lost over time through symmetric differentiation (terminal division), only a minority of such clones persist and expand in size. A newly arisen clone carrying a neutral mutation, which although not altering cell behavior might facilitate neoplastic progression is likely to be shed from the tissue within a few rounds of division (**Fig. 4**, clone marked with X). By chance, only a small proportion of clones that carry neutral mutations will persist long term, so the acquisition of *multiple* mutations in a single clone is an even more remote possibility.^24^ However, a mutation that tilts progenitor cell fate toward proliferation by increasing the probability of divisions producing 2 progenitor cells is much more likely to create a dominant and persistent clone which may undergo further mutations and progress toward cancer.

**Figure 2:**
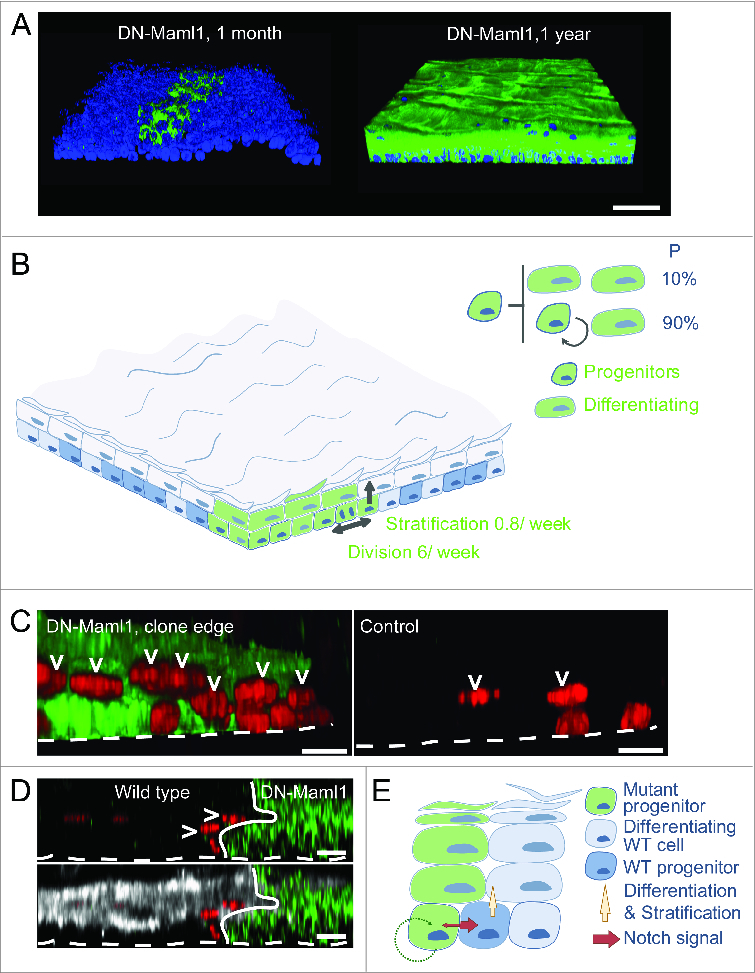
(**See previous page**). Notch inhibition confers clonal dominance. (**A**) Side views of 3-dimensional reconstructions of confocal z stacks showing clonal areas of wholemounts of esophageal epithelium immunostained for DN-Maml1 (green) at one month and 1 year post induction. Dapi is blue, scale bars 500 μm. (**B**) Effect of clonal DN-Maml1 expression on progenitor cell dynamics. At early time points DN-Maml1 expression (green) increases the rate of progenitor cell division and decreases the rate of differentiating cell stratification. In addition, divisions resulting in 2 differentiating cells are absent, blocking clone loss by differentiation. In combination these changes result in exponential clonal expansion in a background of wild type cells (blue). (**C**) Notch inhibition induces differentiation of adjacent wild type cells. Side view of 3-dimensional reconstructions showing typical appearances of DN-Maml1 induced and uninduced age-matched control epithelial wholemounts. Progenitor cells were labeled with a pulse of Ethinyl deoxy Uridine (EdU, red), taken up by progenitors that were in S phase 48 hours before staining. At the boundary of a DN-Maml1 clone (green), an increased proportion of non-mutant suprabasal EdU+ cells (arrowed) is seen compared with controls, indicative of an increased rate of progenitor differentiation. Dotted line indicates basement membrane, scale bars 10 μm. (**D**) XZ cross sections of a wholemount confocal z-stack from DN-Maml1 induced treated with EdU as described in (**C**). Accelerated stratification at the wild type edge shows typical markers of esophageal differentiation. GFP green, EdU is red and differentiation marker Keratin 4 white, dotted line indicates basement membrane, arrows suprabasal EdU positive cells. Scale bars 10 μm. (**E**) Model of wild type cell elimination through competition with Notch mutant cells. Notch signaling is activated preferentially in wild type cells at the clonal edges due to inhibition of Notch pathway in mutant cells. This prompts stratification and differentiation of wild type progenitors. Clone expansion is accelerated by the active expulsion of wild type cells through differentiation.

**Figure 3.**
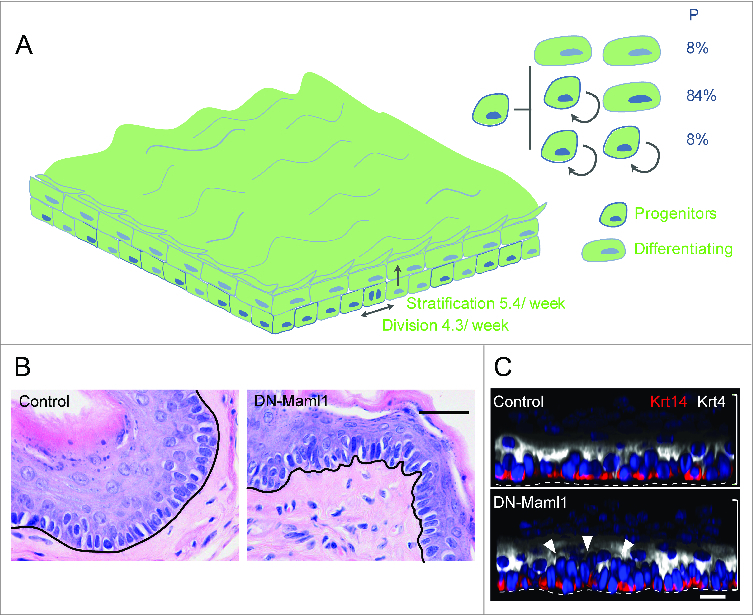
Cell dynamics after complete epithelial replacement by DN-Maml1 cells. (**A**) At long time points after induction, the entire esophageal epithelium is replaced by DN-Maml1 mutant cells (green). As this happens, the 3 division outcomes of normal progenitor cells are reinstated, with balanced probabilities. Tissue turnover is still accelerated, but a new ‘steady-state’ is reached. (**B**) Section of mouse esophageal epithelium showing epithelial buckling at 1 year post-induction in *DM-Maml1* mice compared to aged-matched uninduced controls. Scale bar 20 μm. (**C**) Side view of 3-dimensional reconstructions of confocal images showing increased cell density (arrows) one year post induction in DM-Maml1 mutant epithelium compared to aged-matched uninduced controls. Basal cell marker Keratin 14 is red, suprabasal marker Keratin 4 in white, and Dapi blue. Dotted line indicates basement membrane, brackets indicate epithelial thickness. Scale bar 10 μm.

**Figure 4.**
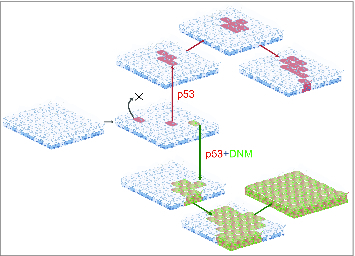
A cellular mechanism of field change. In carcinogen exposed tissues mutations in genes such as p53 (red) are frequent. However, stochastic differentiation leads to most such mutant clones being shed from the epithelium (marked X). However, if a p53 mutant cell is subject to a Notch inhibiting mutation, it achieves clonal dominance. Over time the double mutant clone expands to colonize a large area, resulting in a region of epithelium at increased risk of malignant transformation as it acquires further mutations.

Lineage tracing of mutant cells is a powerful technique with which to define how mutations alter progenitor cell behavior at a qualitative and quantitative level.^34-36^ Candidate mutations that may alter progenitor fate in EE include those affecting the Notch pathway. Notch is a transmembrane receptor, that is cleaved by gamma secretase when it binds its ligand.^37^ This releases the cytoplasmic domain of the protein (Nicd), which migrates to the nucleus where it binds to a multiprotein complex that includes the DNA binding protein Rbpj and Mastermind like 1, Maml1, activating the transcription of Notch target genes.^38^

Multiple lines of evidence point to a role for Notch in squamous and esophageal carcinogenesis.Notch receptors are frequently inactivated by mutation in tumors of squamous epithelium.^39-45^ In keratinocytes, Notch activation drives differentiation, while in the squamous epithelium of the epidermis, loss of *Notch* promotes tumor formation.^46-49^ Motivated by these observations, we performed lineage tracing of esophageal progenitors expressing a dominant negative mutant of Mastermind like 1 (*DN-Maml1*) which blocks Notch signaling by preventing Nicd-induced transactivation. *DN-Maml1* blocks Notch target gene induction and phenocopies the effects of Notch deletion in a range of tissues.^47,50-53^ Crucially, in this model, the *Maml1* mutant is fused to GFP and targeted conditionally to a ubiquitous locus, allowing mutant cells to be visualized using confocal microscopy following cell labeling.

Expression of *DN-Maml1* in individual esophageal progenitors confers a strong competitive advantage on the mutant cells, which generate clones that expand rapidly over the weeks following induction (**Fig. 2A**).^50^ Quantifying clone size at early time points by 3D imaging reveals mutant clones contain several fold more cells than control clones expressing a fluorescent protein reporter. In addition the proportion of differentiated cells is reduced in the mutant clones. Most significantly, by 10 d after induction, a substantial number of control clones are found ‘floating’ in the suprabasal layers after all their progenitor cells have undergone terminal division, generating 2 differentiated cells.^50^ In contrast there are no floating mutant clones, indicating that inhibition of Notch signaling has blocked ‘terminal’ divisions generating 2 differentiating cells. Therefore, expression of *DN-Maml1* renders clones functionally ‘immortal’, as they can no longer be lost by shedding.

Quantitative analysis of mutant clone sizes reveals how *DN-Maml1* expression alters cell behavior soon after induction (**Fig. 2B****)**.^50^ Mutant cells divide 3 fold faster than wild type cells, and, on average, each cell division produces an excess of progenitors over differentiated cells due to the lack of the terminal differentiated division outcome. The differentiated cells that are produced leave the basal layer at a reduced rate. In combination these changes confer a decisive advantage over wild type cells, with mutant clones expanding exponentially. Analysis of gene expression in mutant cells reveals alterations in transcripts implicated in keratinocyte differentiation and cytoskeletal organization consistent with the changes in cell dynamics. For example the stress induced keratin, *Krt6*, is strongly induced in mutant cells. Intriguingly, the differentially expressed genes include the transcription factor *Sox9*, a *Notch* target which is down regulated in mutant clones.^50,54^ *Sox9* is implicated in stem cell regulation in a range of developing and adult tissues including foregut endoderm, and the esophagus, acting to alter the microenvironment via Tgfβ family protein signaling.^55,56^ ^57,58^

These results reveal the intrinsic molecular and cellular characteristics of cells expressing DN-Maml1. However studies in Drosophila indicate some super competitor mutations exert a ‘bystander effect’, actively eliminating wild type cells. For example, winner cells with higher levels of *dMyc* have both cell intrinsic advantages in metabolism and proliferation and secrete factors which induce apoptosis in wild type losers.^6,7,59,60^ In the esophageal epithelium, short term lineage tracing reveals the wild type progenitor cells immediately adjacent to mutant clones stratify at a higher rate than cells distant from the clone **(****Fig. 2C and** **D****)**.^50^ The morphology and expression of markers such as Krt4 suggests the stratifying wild type cells are undergoing a normal process of differentiation (**Fig. 2D**). Increased differentiation of wild type cells has also been observed in co-cultures of wild type and Notch inactivated human keratinocytes in culture.^46^

What is the mechanism driving increased wild type cell stratification at *DN-Maml1* clone margins? The observation that increased differentiation only occurs in wild type cells in contact with mutant clones argues that cell-cell signaling promotes differentiation. One candidate pathway is the Notch itself, as imbalances in Notch signaling between adjacent cells can alter cell fate of in a variety of epithelia.^61,62^ In EE, treatment with a gamma secretase inhibitor which blocks Notch signaling restores the stratification rate of wild type cells at the *DN-Maml1* clone edge to normal. This argues that a Notch mediated bystander effect contributes to the super competitor phenotype of *DN-Maml1* clones (**Fig. 2E**).

Eventually, the active expulsion of wild type cells via differentiation induced stratification, combined with exponential expansion of mutant clones leads to the entire epithelium being replaced by mutant cells (**Fig. 2A**).^22^ What is surprising, given the dynamics of mutant cells at early time points, is that a year or more after induction of *DNMaml1* mice are healthy and free from esophageal tumors. At late time points, no wild type cells remain. The mutant epithelium establishes a new steady state and retains its integrity. Lineage tracing within the mutant epithelium reveals while progenitors still have increased rates of division and stratification compared to wild type cells the terminal division outcome is reinstated and, the balanced production of progenitors and differentiating cells restored (**Fig. 3A**). The mechanism of this rebalancing is not known, but it may be significant that once wild type cells have been lost, the mutant epithelium becomes crowded, with a 30% increase in the density of basal cells and buckling of the epithelium, consistent with increased mechanical stress^50,63^ (**Fig. 3B and C**).^50,63^ These changes are reminiscent of epidermis exposed to ultraviolet light, where *p53* mutant clones expand exponentially until they reach several thousand cells in size, when crowding occurs and expansion slows.^19,64^ In culture, crowding of keratinocytes promotes their differentiation followed by stratification out of the basal layer.^65^ Differentiation is functionally equivalent to the density dependent cell extrusion widely observed epithelial tissues.^66-68^ Linking cell density to differentiation offers a robust defense against any mutation causing increased cell production and may result from the increased mechanical pressure created by an expanding clone within an epithelium.^63^

If EE can tolerate *Notch* inhibiting mutations so effectively, does *Notch* mutation play a significant role in early carcinogenesis? A carcinogen exposed epithelium may contain numerous cells carrying oncogenic mutations, such as in *p53,* which will form mutant clones. However, unless probability of cell loss by differentiation and shedding is significantly reduced, most of these clones will not persist for sufficient time to acquire additional mutations.^20,36^ If a Notch inhibiting mutation occurs in a cell carrying a preexisting mutation, it might confer a super competitor phenotype, creating a field change within which carcinogenesis can progress (**Fig. 4**). Indeed, following single cell induction of *DN-Maml1* in nitrosamine treated mice carrying sporadic *p53* mutant clones, rare double mutant clones are observed. These are much larger than clones carrying *p53* mutations alone, and are similar in size to *DN-Maml1* clones in the same animals. When such animals are aged, large regions of double mutant epithelium are seen, illustrating how *DN-Maml1* clonal dominance can be hijacked by other less competitive mutations to colonize the tissue. This also exemplifies how oncogene cooperation may occur via cell dynamics as well as at the level of intracellular signaling and transcription.

Once *DN-Maml1* mutant areas have been established in nitrosamine treated mice, they have a several fold higher incidence of tumor formation/unit area than adjacent wild type regions in the same animals, and the lesions formed are significantly larger than those arising from wild type regions. This argues Notch inhibiting mutations promotes esophageal tumor formation beyond conferring clonal dominance, as has been shown in the epidermis.^49^ Loss of Notch in the epidermis is strongly linked with the induction of systemic immune changes and stromal inflammation.^48,69,70^ Interestingly there is no evidence of epithelial, stromal or systemic immune changes following *DN-Maml1* expression in the esophagus, likely reflecting organ specific tuning of the immune system.

These observations suggest a cellular mechanism for the phenomenon of ‘field change’, observed in carcinogen exposed human epithelia. Areas of apparently normal epithelium have been shown to harbor clonal mutations and generate multiple dysplastic lesions and tumors over time.^20,21,71^ In squamous epithelium there is no constraint to clone expansion, so a single mutant progenitor whose dynamics are altered in a similar fashion to that produced by *DNMaml* may colonize a large region.^23-25^ Over a prolonged period of carcinogen exposure an epithelium may become a patchwork of clonal fields each carrying a super competitor mutation and any preceding oncogenic genomic alterations present in the founder cell. Understanding how the behavior of cells within these mutant fields changes as they acquire additional genetic damage will be key designing rational strategies to decrease cancer risk in humans with a large burden of mutations.^72^

In conclusion, the findings reviewed here argue Notch should be added to the expanding list of pathways implicated in cell competition. The ability of Notch inhibited progenitor cells to drive out wild type neighbors and replace an entire tissue compartment places Notch in the class of super competitors. The colonization of the esophageal stem cell niche parallels the effect of ‘winner’ *Wnt* pathway, *KRas* mutations in intestinal epithelium and *p53* mutation in transplanted haematopoietic stem cells.^36,73,74^ In the context of early cancer development, where cells may harbor multiple mutations, such mutants play a crucial role in immortalizing and expanding clones carrying oncogenic genome alterations. If increased cell density promotes cell loss by differentiation, tissues may re-establish cell fate balance and preserve their functional integrity. However, such resilience results in carcinogen exposed epithelia becoming a patchwork of super-competitor mutations in which cancer evolution will continue unless mutagen exposure ceases.^20^
